# Echocardiographic assessment of intimal thickness growth of patent ductus arteriosus in neonates and analysis of influencing factors

**DOI:** 10.1007/s10554-022-02531-0

**Published:** 2022-02-02

**Authors:** Xin-Lu Hu, Hui Wang, Cui Hou, Miao Hou, Shi-Hong Zhan, Tao Pan, Yue-Yue Ding, Pei-Pei Gu, Qiu-Qin Xu

**Affiliations:** 1 Department of Pediatric Cardiology, Children’s Hospital of Soochow University, 92 Zhongnan Road, Suzhou, 215003 Jiangsu China; 2 Department of Neonatology, Children’s Hospital of Soochow University, Suzhou, Jiangsu China

**Keywords:** Echocardiography, Intimal thickness, Neonates, Patent ductus arteriosus

## Abstract

The spontaneous closure rate of patent ductus arteriosus (PDA) is high, and the necessity of early intervention is debated. Quantitative echocardiographic assessment of the intima in PDA has not been reported. This study evaluated intimal thickness growth in neonatal cases of PDA via echocardiography and investigated its correlation with clinical factors. Seventy-three neonates were enrolled, and echocardiography was performed three times: within 24 h post-birth (first echo), 48 h after the first echo (second echo), and before discharge (third echo). According to PDA outcome, the neonates were divided into the PDA-open group (n = 18 cases), PDA-closure at second echo group (n = 32 cases), and non-PDA at first echo group (n = 23 cases). We measured the intimal thickness (IT1 and IT2 at first and second echo, respectively), lumen diameter of ductus arteriosus (D1 and D2 at first and second echo, respectively), IT1/D1 ratio, and intimal thickness growth rate (V). Correlations between echocardiographic indicators, perinatal factors, and clinical treatment were analyzed. On first echo, the PDA-open group showed a significantly lower IT1/D1 than the combined PDA-closure group (P < 0.05). On second echo, the PDA-open group showed a significantly lower IT2 and V than the PDA-closure group as well as a significantly higher D2 (P < 0.05). Smaller gestational age correlated with a larger D2 but smaller IT2 and V (P < 0.05) and a higher level of respiratory support within 72 h post-birth correlated with a larger D2 and smaller IT 2 (P < 0.05). Increasing oxygen demand within 72 h of birth correlated with a larger D1 and D2 (P < 0.05). Echocardiographic assessment of intimal thickness growth in PDA may provide an approach for predicting spontaneous PDA closure, thereby guiding decision-making regarding early intervention.

**Supplementary Information:**

The online version contains supplementary material available at 10.1007/s10554-022-02531-0.

## Introduction

The ductus arteriosus (DA) connects the aorta and the main pulmonary artery in the fetal circulation, and spontaneous functional closure typically occurs in healthy neonates within 72 h after birth. Permanent closure of the DA includes functional closure caused by muscle contraction and anatomical closure achieved by morphological and molecular remodeling. The process of DA closure is complex and effected by many factors, and the mechanism remains incompletely understood. Research has shown that after the second trimester, the placenta produces a high concentration of the vasodilator prostaglandin E2 (PGE2) that acts to maintain DA expansion during the subsequent fetal period [1]. About 10–15 h after delivery, the DA functionally closes due to the increase of arterial oxygen partial pressure (PaO_2_), a sharp decrease in the circulating PGE2 level, a decrease in the blood pressure in the DA lumen, and a decrease of the number of PGE2 receptors on the DA tube wall [2]. Research has also shown that anatomical closure of the DA begins in the second trimester and progresses throughout the remaining period of fetal development, continuing for weeks to months after delivery [3]. A series of histological changes include the deposition of extracellular matrix under the endothelium, disintegration of the inner elastic layer, a loss of elastic fibers in the media, and migration of smooth muscle cells in the media. During the occlusion of the lumen, the intima and media gradually became elastic and collagenous. Luminal fibrosis gradually obstructs the lumen in the weeks to months following anatomical closure, resulting in a central collagenized and locally calcified ligamentum arteriosus [4–6].

Abnormality of the process of DA closure results in patent ductus arteriosus (PDA), which accounts for 10–21% of congenital heart disease and is significantly more common in premature infants than full-term infants [4, 7]. Because the spontaneous closure rate of PDA is high, the need for early intervention is debated. Moreover, the most appropriate approach to early treatment and expectant therapy remains to be determined. For preterm infants specifically, it is still unclear which method is most beneficial, but many studies have reported the value of non-selective cyclooxygenase (COX) inhibitors for DA closure in preterm infants with PDA. Still some studies have shown that drug intervention did not provide a superior benefit compared with conservative treatment [8–11]. Therefore, a method to predict the likelihood of spontaneous DA closure and thus the need for early intervention would support the successful treatment of neonates with PDA.

**Fig. 1:**
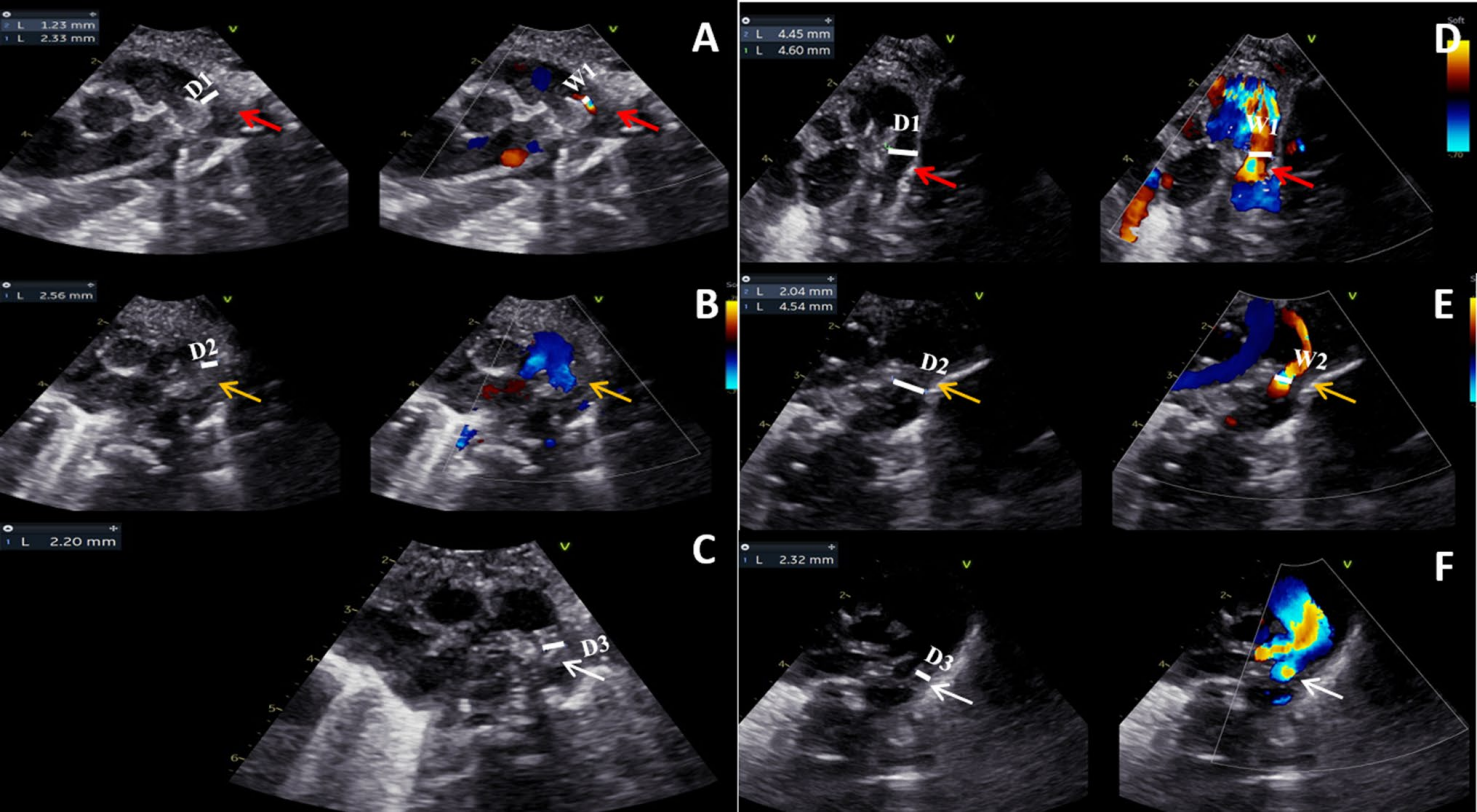
Intima growth in two representative cases of PDA. **A**–**C** Images from a neonate in the PDA-closure group at the first, second and third echo; **D**–**F** images from a neonate in the PDA-open group at the first, second and third echo. **A** Within 24 h of birth, the intima appeared as a hypoechoic, uneven and loosely formed structure (red arrow); **B** within 48–72 h of birth, the lumen was occluded with thickened intima, and the echo and thickness of the intima became even (yellow arrow); **C** at 26 days of age, an arterial ligament was formed (white arrow); **D** within 24 h of birth, the intima was very thin (red arrow), and the shunt passing through the DA was wide; **E** within 48–72 h of birth, the intima was thickened (yellow arrow),the shunt passing through the DA became thinner; **F** at 21 days of age, the DA was contracted, the lumen was occluded, and the intima was still hypoechoic (white arrow). *D1* lumen diameter at first echo, *W1* width of transcatheter flow bundle at first echo, *D2* lumen diameter at second echo, *W2* width of transcatheter flow bundle at second echo, *D3* lumen diameter at third echo

The traditional evaluation criteria for PDA include the anatomical shape, diameter, and length of the DA on two-dimensional echocardiography as well as relevant characteristics on color Doppler echocardiography [12]. Using echocardiography, we previously observed a slightly hypoechoic intimal structure attached to the inner wall of the DA in neonates, and variation in this structure was observed according to the outcome of the DA (Fig. 1). However, quantitative assessment of the thickness increase of this DA intimal structure based on echocardiography assessment has not been reported in the literature. The purpose of this study was to observe the characteristics of dynamic DA intimal thickness changes by echocardiography in neonates and to identify any correlations between these changes and clinical indicators. Further, we aimed to provide insight regarding a method for predicting spontaneous DA closure, which would help determine the value of early intervention in neonates with PDA.

## Materials and methods

### Study population

A total of 73 neonates (37 males [50.7%]; mean gestational age, 35.4 ± 3.6 weeks) admitted to the Department of Neonatology of Children’s Hospital Affiliated of Soochow University from July 2020 to April 2021 were enrolled as the study population. Neonates hospitalized within 24 h of birth were included in the study. The study population consisted of 26 full-term neonates and 47 preterm neonates (including 15 extremely preterm neonates). The incidence of pulmonary hypertension was 21.9%. The exclusion criteria included: atrial shunt over 5 mm, other congenital heart diseases, arrhythmia, congenital malformations of other systems, severe infection, death before discharge, and abandonment of treatment.

### Research methods

Perinatal, clinical and echocardiographic data were collected. Three echocardiographic examinations were performed: the first within 24 h of birth (first echo), the second at 48 h after the first echo (second echo), and the third before discharge (neonatal age of 7–29 days, median age of 14 days).

The following perinatal data were collected: gestational age at birth, birth weight, body surface area (BSA) at birth, sex, single or multiple pregnancy, maternal eclampsia or preeclampsia, application of antenatal corticosteroids, hematocrit (HCT) within 24 h after birth, and complications such as neonatal asphyxia, neonatal respiratory distress syndrome (NRDS) or respiratory failure. The diagnostic criteria for NRDS were PaO_2_ < 50 mmHg, central cyanosis, oxygen inhalation to maintain PaO_2_ > 50 mmHg, and typical chest radiographs [13]. There is no uniform standard currently for the diagnosis of neonatal respiratory failure. In this study, the diagnoses was based on clinical symptoms such as three depressions sign, groan, central cyanosis, refractory apnea, respiratory rate > 60 beats/min, combined with laboratory indicators including PaCO_2_ > 60 mmHg, PaO_2_ < 60 mmHg or oxygen saturation < 80% at FiO_2_ 100%, pH < 7.25.

The following clinical data were collected: postnatal use of pulmonary surfactant (PS), the highest degree of oxygen therapy within 72 h after birth (non-oxygen, hood oxygen or mechanical ventilation), whether oxygen demand increased within 72 h after birth (upgrade of type of oxygen therapy or oxygen concentration), use of furosemide within 72 h after birth, and ibuprofen use.

Echocardiography was performed using a Vivid E90 instrument (GE Vingmed Ultrasound, Horten, Norway) with a 2.9–5.8 MHz phased-array transducer. M-mode measurements included left ventricular end-diastolic diameter (LVEDd1 and LVEDd2 at first and second echo, respectively) and left ventricular ejection fraction (EF1 and EF2 at first and second echo, respectively).

**Fig. 2:**
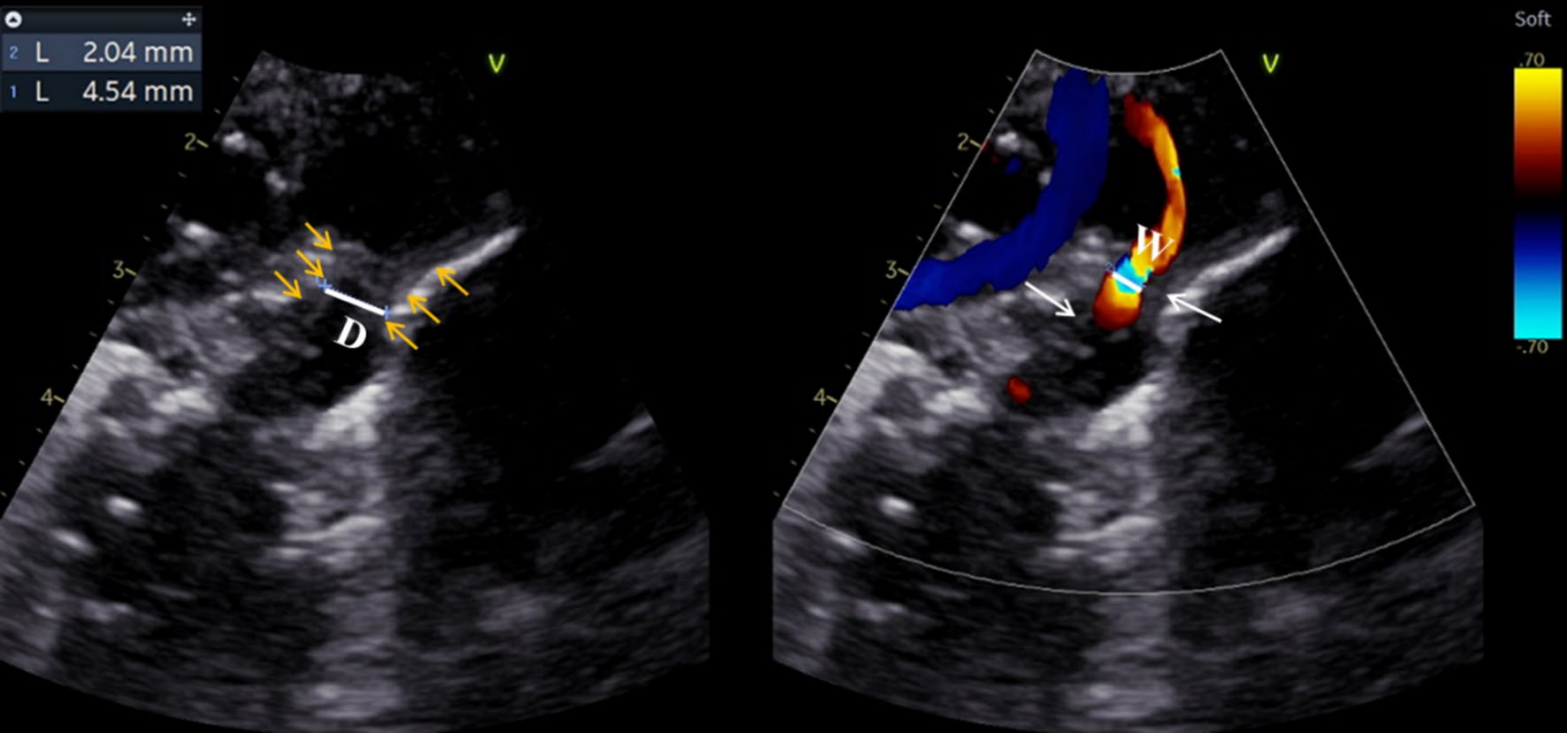
Imaging and measurement of the intima in a representative case of PDA. Caliper 1: Lumen diameter (D); Caliper2: The width of the transcatheter flow bundle (W); Yellow arrows: inner margin of the wall of PDA; White arrows: intima of PDA, appearing as hypoechoic, uneven

The dynamic images of PDA were obtained based on the long axis view of PDA at the superior sternal fossa, which shows the left pulmonary artery, the right pulmonary artery, and the long axis of PDA (the three-finger view). Two-dimensional gain was adjusted until the inner wall and intima of DA were clearly displayed, and color Doppler gain were adjusted to ensure complete filling but no overflow of the shunt flow of DA. The recorded parameters of PDA included: lumen diameter (D1, D2 and D3 at the first, second and third echo, respectively), intimal thickness (IT1, IT2 and IT3 at the first, second and third echo, respectively), diameter of aortic end of the ampulla (DO1 and DO2 at first and second echo, respectively), the intimal thickness to lumen diameter ratio at first echo (IT1/D1), and the growth rate of intimal thickness between the first and second echo and between the second and third echo (Va and Vb, respectively). For measurement of parameters of PDA, the simultaneous mode was used on Echopac. The dynamic images were played back during the ventricle systole to the frame in which the flow bundle and the intima were best displayed. At the same level at the narrowest point of the DA, D was measured from one inner edge of the DA tube wall to the other inner edge, and the width of the transcatheter flow bundle (W) was measured exactly parallel to D (Fig. 2). Then IT = (D−W)/2. DO was measured at the aortic end of the ampulla. Measurements were performed by two experienced sonographers. Mean values were obtained from measurements repeated 3 times. V was defined as the average growth rate of PDA intimal thickness every 24 h between two echocardiographic examinations, where the number of days between the second and third echo was t. Thus, Va = (IT2−IT1)/2 and Vb = (IT3−IT2)/t.

### Grouping

According to PDA outcome, the neonates were divided into three groups: the PDA-open group, in which PDA was present at first and second echo; the PDA-closure group, in which PDA was present at the first echo and the PDA was closed at the second echo; and the non-PDA group, in which the DA was closed at first echo.

### Statistical analysis

Statistical analysis was conducted using SPSS version 23.0 (SPSS Inc., Chicago, IL, USA.) Clinical data were compared among the three patient groups using the Chi-square test or Kruskal–Wallis H rank-sum test, as appropriate. Echocardiographic indicators were compared between the PDA-open and PDA-closure groups using Student’s t-test or Wilcoxon rank-sum test, as appropriate. The Cochran–Mantel–Haenszel test (CMH) was used to examine the difference in the highest degree of oxygen therapy within 72 h between the PDA-open and PDA-closure groups. Analysis of variance (ANOVA) or Kruskal–Wallis H rank-sum test was used to compare PDA parameters in neonates that received different oxygen therapies. Bonferroni correction was used for multiple comparisons. Due to the small number of cases, Fisher’s exact probability method was used to detect differences in PDA outcome between infants treated with or without ibuprofen. Spearman correlation analysis was applied to identify correlations between PDA parameters and clinical data. Inter- and intra-observer variabilities were assessed in 10 randomly selected neonates using Bland–Altman analyses. A two-tailed P value of less than 0.05 was considered statistically significant.

## Results

### Clinical data of neonates according to DA closure outcome

The gestational age at birth was significantly lower for the PDA-open group than the PDA-closure and non-PDA groups, but did not differ significantly between the PDA-closure and non-PDA groups. BSA at birth was significantly lower in the PDA-open group than in the non-PDA group, but did not differ significantly between the PDA-open and PDA-closure groups. The proportions of patients with neonatal asphyxia in the PDA-open and PDA-closure groups was significantly higher than that in the non-PDA group. The proportions of patients with NRDS or respiratory failure in the PDA-open group was higher than that in the non-PDA group, but no difference was found between the PDA-open and PDA-closure groups. There were no statistical differences among the three groups in birth weight, sex, multiple pregnancy, maternal eclampsia or preeclampsia, prenatal application of glucocorticoid, or HCT within 24 h after birth. Additionally, no statistical differences in the PS application rate and furosemide application rate within 72 h were observed between the PDA-open and PDA-closure groups (Table 1).

**Table 1:** Clinical data of neonates in the PDA-open, PDA-closure and non-PDA groups

	PDA-open group	PDA-closure group	Non-PDA group	χ^2^ (Kruskal–Wallis H)	P
	(n = 18)	(n = 32)	(n = 23)		
Gestational age (weeks)	32.6 ± 3.7	35.9 ± 3.2^a^	36.8 ± 3.1^a^	(13.896)	0.001
Birth weight (g)	2035.0 ± 1018.6	2386.6 ± 806.0	2510.0 ± 777.3	(5.945)	0.051
BSA (m^2^)	0.13 ± 0.04	0.15 ± 0.04	0.16 ± 0.04^a^	7.097	0.029
Male sex	10 (55.6)	15 (46.9)	12 (52.2)	0.377	0.871
Multiple pregnancy	3 (16.7)	4 (12.5)	1 (4.3)	1.760	0.402
Maternal eclampsia or preeclampsia	6 (33.3)	11 (34.4)	3 (13.0)	3.484	0.195
Prenatal application of glucocorticoid	12 (66.7)	15 (46.9)	8 (34.8)	4.139	0.136
Neonatal asphyxia	14 (77.8)	22 (68.8)	6 (26.1)^a,b^	13.976	0.001
NRDS or respiratory failure	8 (44.4)	7 (21.9)	1 (4.3)^a^	9.486	0.009
HCT (%)	0.480 ± 0.076	0.503 ± 0.061	0.498 ± 0.090	(2.604)	0.272
PS application	4 (22.2)	2 (6.3)	–	1.476	0.225
Furosemide application	4 (22.2)	5 (15.6)	–	0.040	0.842

Data are expressed as mean ± standard deviation (SD) or number (percentage)

^a^P < 0.05 compared with PDA-open group

^b^P < 0.05 compared with PDA-closure group

### Comparison of echocardiographic indicators between neonates with PDA according to DA closure

No significant differences in LVEDd1, EF1, D1, DO1, IT 1, LVEDd2, and EF2 were observed between the PDA-open and PDA-closure groups. The IT1/D1 was lower in the PDA-open group than in the PDA-closure group, and D2 and DO2 were significantly higher in the PDA-open group than in the PDA-closure group. Additionally, IT2 and Va were significantly lower in the PDA-open group than in the PDA-closure group (Table 2).

**Table 2:** Echocardiographic indicators among neonates in the PDA-open and PDA-closure groups

	PDA-open group	PDA-closure group	t (Wilcoxon W)	P
	(n = 18)	(n = 32)		
LVEDd 1 (mm)	17.2 ± 2.5	17.1 ± 2.0	0.132	0.896
EF1 (%)	65.3 ± 6.0	68.3 ± 4.8	− 1.876	0.067
D1 (mm)	2.8 ± 0.9	2.4 ± 0.5	1.832	0.080
DO1 (mm)	3.0 ± 0.9	2.7 ± 0.8	(754.000)	0.210
IT1 (mm)	0.09 ± 0.11	0.19 ± 0.18	(366.000)	0.053
IT1/D1	0.03 ± 0.04	0.08 ± 0.08	(354.000)	0.029
LVEDd2 (mm)	17.3 ± 2.0	17.1 ± 1.9	0.333	0.740
EF2 (%)	69.1 ± 8.2	70.2 ± 5.7	− 0.563	0.576
D2 (mm)	2.3 ± 0.8	1.4 ± 0.4	4.656	< 0.001
DO2 (mm)	2.5 ± 1.2	1.8 ± 0.6	(692.000)	0.012
IT2 (mm)	0.16 ± 0.13	0.69 ± 0.20	− 10.127	< 0.001
Va (mm/24 h)	0.04 ± 0.05	0.26 ± 0.13	− 8.485	< 0.001

Data are expressed as mean ± SD

*LVEDd1 and LVEDd2* left ventricular end-diastolic diameter at first and second echo, respectively, *EF1 and EF2* left ventricular ejection fraction at first and second echo, respectively, *D1and D2* lumen diameter at first and second echo, respectively, *DO1 and DO2* diameter of aortic end of the ampulla at first and second echo, respectively, *IT1 and IT2* intimal thickness at first and second echo, respectively, *IT1/D1* the intimal thickness to lumen diameter ratio at first echo, *Va* the growth rate of intimal thickness between the first and second echo

### Oxygen therapy for neonates with PDA

Comparison of the highest degree of oxygen therapy within 72 h between neonates in the PDA-open and PDA-closure groups.

Within 72 h after birth, the neonates in the PDA-open group were most commonly treated with mechanical ventilation, followed by hood oxygen. The proportions of neonates in the PDA-closure group treated with the three methods of oxygen therapy were roughly equal, and the difference in the application of different forms of oxygen therapy between the two groups was statistically significant (Table 3).

**Table 3:** Comparison of the highest degree of oxygen therapy within 72 h between the PDA-open and PDA-closure groups

	Cases	Non-oxygen	Hood oxygen	Mechanical ventilation
PDA-open group	18	1 (5.6)	5 (27.8)	12 (66.7)
PDA-closure group	32	11 (34.4)	10 (31.3)	11 (34.4)
χ^2^		6.644		
P		0.036		

Data are expressed as number (percentage)

### Comparison of PDA parameters in neonates that received different oxygen therapies

The D2 and DO2 were higher in neonates that received mechanical ventilation than in those that did not receive oxygen, and DO2 was higher in neonates that received mechanical ventilation than in those that received hood oxygen. D2 and DO2 did not differ significantly between neonates who did not receive oxygen and those that received hood oxygen. Furthermore, no significant differences in D1, DO1, IT1 /D1, IT1, IT2 and Va were observed among the three groups based on oxygen therapy (Table 4).

**Table 4:** Comparison of PDA parameters in neonates that received different oxygen therapies

	No oxygen	Hood oxygen	Mechanical ventilation	F (Kruskal–Wallis H)	P
	(n = 12)	(n = 15)	(n = 23)		
D1 (mm)	2.41 ± 0.56	2.25 ± 0.48	2.78 ± 0.84	3.000	0.559
D2 (mm)	1.38 ± 0.41	1.53 ± 0.43	2.01 ± 0.89^a^	4.414	0.018
DO1 (mm)	2.70 ± 0.71	2.51 ± 0.70	2.96 ± 0.92	1.411	0.254
DO2 (mm)	1.60 ± 0.40	1.73 ± 0.54	2.46 ± 1.15^a,b^	5.160	0.009
IT1 (mm)	0.18 ± 0.20	0.19 ± 0.15	0.11 ± 0.15	(3.434)	0.180
IT2 (mm)	0.61 ± 0.26	0.58 ± 0.32	0.39 ± 0.31	(5.493)	0.064
Va (mm/24 h)	0.22 ± 0.15	0.20 ± 0.16	0.14 ± 0.15	1.272	0.290
IT1/D1	0.73 ± 0.75	0.82 ± 0.60	0.48 ± 0.75	(4.187)	0.123

Data are expressed as mean ± SD

^a^P < 0.05 compared with no oxygen group

^b^P < 0.05 compared with hood oxygen group

*D1and D2* lumen diameter at first and second echo, respectively, *DO1 and DO2* diameter of aortic end of the ampulla at first and second echo, respectively, *IT1 and IT2* intimal thickness at first and second echo, respectively, *Va* the growth rate of intimal thickness between the first and second echo, *IT1/D1* the intimal thickness to lumen diameter ratio at first echo

### Repeatability and reproducibility of intimal thickness

**Fig. 3:**
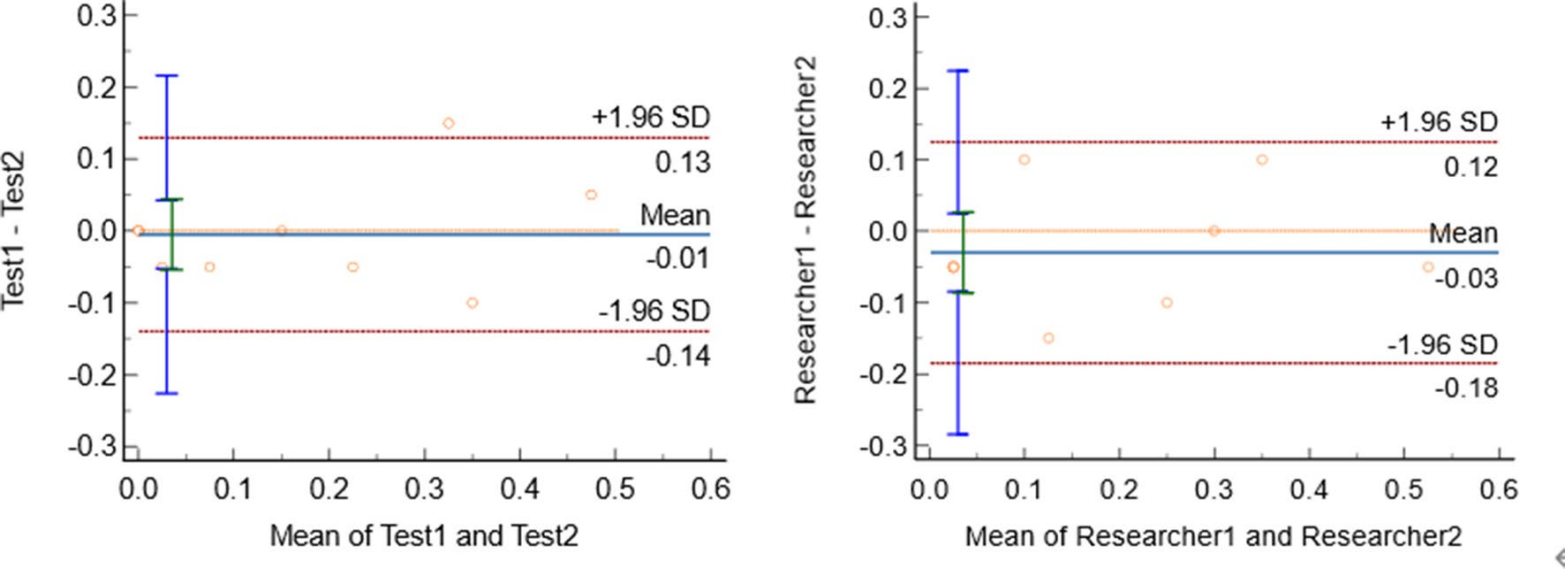
Bland–Altman analysis of inter-and intra-observer variability for intimal thickness. Blue lines represent bias, and red dotted lines represent 95% limits of agreement for measurements performed in 10 patients

The data for inter-and intra-observer variability of intimal thickness are shown in Table 5. Our analyses showed good repeatability and reproducibility for intimal thickness measurement on echocardiography (Fig. 3).

**Table 5:** Repeatability and reproducibility of intimal thickness measurement

	Mean ± SD	Mean ± SD	Bias	95% confidence interval (bias)	P	95% limits of agreement
Inter-observer variability	0.16 ± 0.13	0.17 ± 0.03	− 0.01	− 0.05 to 0.04	0.83	− 0.14 to 0.13
Intra-observer variability	0.16 ± 0.03	0.19 ± 0.03	− 0.03	− 0.09 to 0.03	0.26	− 0.18 to 0.13

### PDA outcome after treatment with ibuprofen

In the PDA-open group, ibuprofen was used in the cases considered clinically significant PDA, according to the presence of a PDA with clinical signs of an effect on organ function attributable to the DA (such as cardiac murmur, oliguria, Corrigan’s pulse, X-ray showed enlarged heart shadow and increased pulmonary blood). Then 7 of the 18 neonates were given oral ibuprofen suspension to promote PDA closure at 8–16 days of age (median 9 days of age). The course of treatment included a first dose of 10 mg/kg followed by 5 mg/kg at 24 and 48 h after the first dose. At the third echocardiographic examination (16–29 days of age; median age, 18 days), the DA was closed in 5 of 7 cases (closure rate, 71.4%). The difference in PDA outcome before discharge between those who received ibuprofen and those who did not was not statistically significant (Online Resource Table 1).

### Spearman correlation analysis of PDA parameters associated with clinical factors

Spearman correlation analysis showed that D1 was positively correlated with PS, furosemide application and an increase in oxygen demand within 72 h. Additionally, D2 was negatively correlated with gestational age and BSA at birth, and positively correlated with NRDS or respiratory failure, the highest degree of oxygen therapy within 72 h and increased oxygen demand. IT2 was positively correlated with gestational age and birth weight, and negatively correlated with the highest degree of oxygen therapy within 72 h. Va was positively correlated with gestational age and birth weight. No significant correlations between IT1 or the IT1/D1 ratio and various perinatal or clinical indicators were observed. Additionally, there were no significant correlations between ibuprofen use and D3, IT3 and Vb in the PDA-open group (Online Resource Table 2).

## Discussion

In our study, because functional closure of the DA in the non-PDA group had been completed by the first echocardiographic examination, differences in intima thickness growth simply could not be observed in the non-PDA group. Thus, we compared the echocardiographic data of the PDA-open and PDA-closure groups at the first and second echocardiographic examinations. Moreover, to explore the associations between ibuprofen and intimal growth, we only considered echocardiographic DA measurements for the neonates in the PDA-open group at the third echocardiographic examination.

We observed that on two-dimensional imaging, the intima of the DA was a hypoechoic, uneven and loosely formed structure with a clear boundary at the inner DA wall. Its thickness was not uniform throughout the lumen. When the DA was not closed at first echocardiographic examination in the PDA-open and PDA-closure groups, the intima was thin, and even could not be observed in some neonates. Although no statistically significant differences in IT1 and D1 were observed between the PDA-open and PDA-closure groups, the IT1/D1 ratio was lower in the PDA-open group than in the PDA-closure group. This ratio combines intimal thickening and contraction of the DA, making it more representative of the true structural changes of the DA after birth than either the intima or lumen diameter alone. With the thickening of the intima, the lumen was gradually occluded, and the shunt passing through the DA became thinner. Accordingly, the echo and thickness of the intima gradually became even (Fig. 1).

Compared with those in the PDA-open group, the D2 and DO2 were lower and the IT2 was higher in the PDA-closure group, indicating that contraction of the DA and intimal thickening occurred simultaneously, and functional closure of the DA occurred earlier in the PDA-closure group than in the PDA-open group. However, the Va and IT2 in the PDA-open group were significantly lower than those in the PDA-closure group, which could indicate that the mechanism of the anatomical closure was more severely damaged in the PDA-open group than in the PDA-closure group. After the lumen was occluded, we observed that the thickness gradually narrowed and the echo of the intima gradually became stronger, which also indicated fibrosis of the intima and the formation of arterial ligaments on ultrasound (Fig. 1).

D and W were measured at the same level at the narrowest point of the DA during the ventricle systole. However, the intimal thickness was different from the pulmonary artery end to aortic end of the DA, and thus, a slight difference of the measured level could lead to variation. On Echopac, the dynamic images were played back during the systole to the frame in which the flow bundle and the intima were best displayed, and then measurements on different frames of the image would result in inter-observer differences. Our analyses showed good repeatability and reproducibility, indicating that small differences in the measured level or frame have little influence on the results for intimal thickness.

Our data suggest that small gestational age may be an important risk factor for PDA outcome, which is consistent with the conclusions of previously studies [9, 14]. Although no differences in birth weight were observed between groups, the correlation analysis showed that a lower birth weight was associated with smaller IT2 and Va values. Previous studies also showed that the spontaneous closure rate of PDA is lower in infants with a low birth weight [15, 16]. Furthermore, the BSA was higher in the non-PDA group than in the other groups, and the correlation analysis showed that a lower BSA was associated with a larger D2 value. Because small gestational age is often associated with low birth weight and low BSA, the latter two showed the same associations with DA as gestational age. A major limitation of our research is that the proportion of extremely premature cases was small, with only 15 extremely preterm neonates with gestational ages ranging from 27.9 to 31.6 weeks. However, the phenomenon of PDA intima growth was observed by echocardiography in neonates of all gestational ages, which we consider of reference significance for further studies on extremely preterm infants.

Our results showed that D2 was larger in neonates with NRDS or respiratory failure, which is consistent with risk factors for PDA identified in previous clinical studies [15, 17]. The present study also found that sex, multiple pregnancy, maternal eclampsia or preeclampsia, prenatal application of glucocorticoid, neonatal asphyxia, and postnatal HCT within 24 h had no association with the growth of the DA. Conclusions regarding these factors have been inconsistent in previous studies, and future studies with larger sample sizes from multiple centers are needed [9, 17–20].

Previous studies have shown that after the application of PS replacement therapy, preterm neonates with PDA complicated by NRDS showed alveolar dilation and decreased pulmonary resistance, leading to increased left-to-right shunt [21, 22]. Loop diuretics such as furosemide keep the PDA open by stimulating the kidney to synthesize PGE2 [23]. However, Thompson et al. showed that furosemide exposure was not associated with an increased probability of PDA requiring treatment [24]. In the present study, we found no significant difference in PS application and furosemide application rate within 72 h between the PDA-open and PDA-closure groups. The application of PS and furosemide was associated with larger D1. However, we cannot determine causality due to numerous confounders arising from the underlying diseases and clinical treatments of the neonates.

In the present study, neonates in the PDA-open group were more likely to receive mechanical ventilation as respiratory support than those in the PDA-closure group. Both D2 and DO2 were lower in the mechanical ventilation group than in the no-oxygen group, and DO2 was also lower than that in the hood oxygen group. The correlation analysis showed that a higher level of oxygen therapy was associated with a greater D2 and smaller IT2. Animal studies have previously shown that positive end-expiratory pressure (PEEP) can reduce left-to-right shunt and increase systemic circulation [25]. In a clinical study, appropriate PEEP (4–6 cm H_2_O) was beneficial for PDA closure in preterm infants requiring mechanical ventilation [26]. Harkin et al. suggested that mechanical ventilation is independently associated with an increased risk of treatment for PDA [27]. Den Harink et al. also proposed that invasive respiratory support is an important predictor of the requirement of surgical intervention for PDA [28]. Our study suggests that with a higher level of oxygen inhalation needed within 72 h after birth, the condition of DA contraction and intimal thickness growth was worse.

The spontaneous closure rate of PDA is high, and thus, the need for intervention is still debated. Moreover, the choice between early treatment and expectant therapy has not been decided [11]. Non-selective COX inhibitors have been found beneficial for PDA closure in preterm infants. Ibuprofen is a non-selective COX inhibitor that inhibits the pathway by which arachidonic acid produces prostaglandin through COX-2 catalysis and is currently the preferred drug for clinical treatment of PDA [29]. Considering that prostaglandins are extensively involved in the functional and anatomical closure mechanisms of PDA [4], we suppose that ibuprofen might promote the growth of DA intima as a non-selective cyclooxygenase (COX) inhibitor. Then IT3 and Vb were measured in the PDA-open group at the third echocardiographic examination. However, the data showed no association between ibuprofen therapy and the diameter reduction nor the intimal growth in PDA, which suggests that further research on the necessity of ibuprofen intervention is needed. However, our result might have been due to our small sample size.

## Conclusions

We observed the morphological characteristics and growth status of the DA intima through a series of echocardiograms and analyzed its correlation with clinical factors. We found that in the PDA-open group, the ratio of intimal thickness to lumen diameter was lower, the DA intima was thinner, the rate at which the intimal thickness increased was slower, and the lumen diameter was wider. Gestational age was found to be an important factor in DA contraction and intimal thickness growth. Mechanical ventilation was shown to not be conducive to the closure of PDA. Echocardiographic assessment of intimal thickness growth in PDA is valid and may provide an approach for predicting spontaneous closure of the DA and determining the value of early intervention in neonates with PDA.

### Supplementary Information

Below is the link to the electronic supplementary material.

Supplementary file1 (DOCX 15 kb)

## Data Availability

The datasets used and/or analyzed during the current study are available from the corresponding author on reasonable request.

## Abbreviations

PDA: Patent ductus arteriosus
DA: Ductus arteriosus
D: Lumen diameter of ductus arteriosus
IT: Intimal thickness of ductus arteriosus
V: Growth rate of intimal thickness
BSA: Body surface area
NRDS: Neonatal respiratory distress syndrome
HCT: Hematocrit
PS: Pulmonary surfactant
LVEDd: Left ventricular end-diastolic diameter
EF: Left ventricular ejection fraction
DO: Diameter of aortic end of the ampulla
