# Supplementary material for: Echocardiographic assessment of intimal thickness growth of patent ductus arteriosus in neonates and analysis of influencing factors
Source: Int J Cardiovasc Imaging. 2022 Feb 2;38(7):1443–52. doi: 10.1007/s10554-022-02531-0 (PMC11142939; doi:10.1007/s10554-022-02531-0)
Supplement: Supplementary file 1 — Supplementary file1 (DOCX 15 kb) [file 10554_2022_2531_MOESM1_ESM.docx]

**Online Resource Table 1**

PDA outcome according to ibuprofen treatment among neonates in the PDA-open group

|  | Cases | Ibuprofen treatment | No ibuprofen treatment |
| --- | --- | --- | --- |
| PDA closed before discharge | 15 | 5(33.3) | 10(66.7) |
| PDA not closed before discharge | 3 | 2(66.7) | 1(33.3) |
| Fisher exact value | | 0.528 | |
| P | | 0.329 | |

Data are expressed as number (percentage)

**Online Resource Table 2**

Spearman correlation analysis of PDA parameters associated with clinical factors

|  | D1 | D2 | D3 | IT1 | IT2 | IT3 | IT1/D1 | Va | Vb | |
| --- | --- | --- | --- | --- | --- | --- | --- | --- | --- | --- |
| Gestational age | NS | -0.330 | - | NS | 0.439 | - | NS | 0.398 | | - |
| Birth weight | NS | NS | - | NS | 0.317 | - | NS | 0.286 | | - |
| BSA | NS | -0.317 | - | NS | NS | - | NS | NS | | - |
| Male sex | NS | NS | - | NS | NS | - | NS | NS | | - |
| Multiple pregnancy | NS | NS | - | NS | NS | - | NS | NS | | - |
| Maternal eclampsia or preeclampsia | NS | NS | - | NS | NS | - | NS | NS | | - |
| Prenatal application of glucocorticoid | NS | NS | - | NS | NS | - | NS | NS | | - |
| Neonatal asphyxia | NS | NS | - | NS | NS | - | NS | NS | | - |
| NRDS or respiratory failure | NS | 0.305 | - | NS | NS | - | NS | NS | | - |
| HCT | NS | NS | - | NS | NS | - | NS | NS | | - |
| PS application | 0.355 | NS | - | NS | NS | - | NS | NS | | - |
| Furosemide application | 0.369 | NS | - | NS | NS | - | NS | NS | | - |
| Oxygen therapy | NS | 0.354 | - | NS | -0.317 | - | NS | NS | | - |
| Increased oxygen demand | 0.320 | 0.291 | - | NS | NS | - | NS | NS | | - |
| Ibuprofen treatment | - | - | NS | - | - | NS | - | - | | NS |
